# Supplementary material for: Structural dynamics between Argonaute-2 and CK1α promote target RNA release in microRNA-mediated silencing
Source: bioRxiv. 2026 Mar 25:2026.03.23.713669. Preprint. [Version 1] doi: 10.64898/2026.03.23.713669 (PMC13041839; doi:10.64898/2026.03.23.713669)
Supplement: Supplement 1 [file media-1.pdf]

Supplemental figures and tables

Structural dynamics between Argonaute-2 and CK1α promote target RNA release in miRNA-mediated silencing

Ankur Garg[1,2], Leah Braviner[1,3], Armend Axhemi[1,2] , Brianna Bibel[1,3,#] and Leemor Joshua-Tor[1,2,*].

[1] W. M. Keck Structural Biology Laboratory, Cold Spring Harbor Laboratory, One Bungtown Road, Cold Spring Harbor, New York, 11724 USA

[2] Howard Hughes Medical Institute, Cold Spring Harbor Laboratory, One Bungtown Road, Cold Spring Harbor, New York, 11724 USA

[3] School of Biological Sciences, Cold Spring Harbor Laboratory, One Bungtown Road, Cold Spring Harbor, New York, 11724 USA

[#] Current address –Loyola Marymount University, Featherston Life Science Building, 1 LMU Drive, Los Angeles, California, 90045 USA

[*] Corresponding author: leemor@cshl.edu

Supplementary Figure 1-

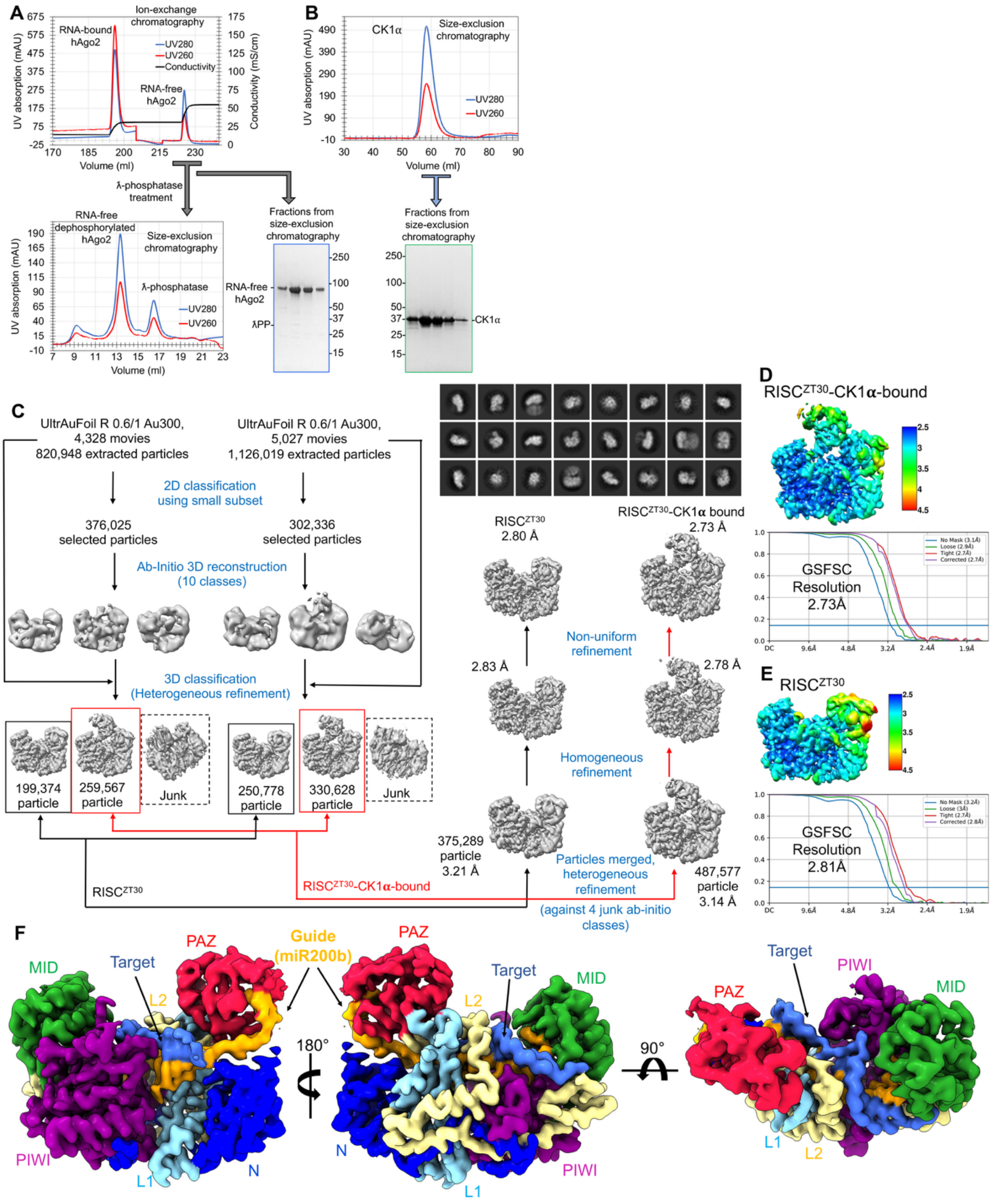

Supplementary Figure 1-

Structural analysis of $RISC^{ZT30}$ in complex with CK1α kinase. (A) Purification of dephosphorylated RNA-free human Ago2 (HsAgo2). The ion exchange chromatogram showing separation of the RNA-bound Ago2 fraction from the RNA-free Ago2 fraction. The SEC chromatogram shows separation of the phosphatase-treated RNA-free Ago2 from the phosphatase. SDS-PAGE showing the purified Ago2 protein used in the study. (B) SEC chromatogram for the CK1α showing a homogeneous protein. The peak fractions analyzed on the SDS-PAGE showing pure protein used in the study. (C) CryoEM data processing workflow for the $RISC^{ZT30}$-CK1α complex. Processed particles from two different datasets were merged to obtain the $RISC^{ZT30}$ and $RISC^{ZT30}$-CK1α structures. The observed 2D class averages for some of the classes used in data processing are also shown. The local resolution estimates and gold-standard FSC (GSFSC) resolution estimate for (D) $RISC^{ZT30}$-CK1α and (E) $RISC^{ZT30}$ maps. (F) CryoEM map for the $RISC^{ZT30}$ in different orientations. Different domains in Ago2, guide, and target are colored and marked.

Supplementary Figure 2-

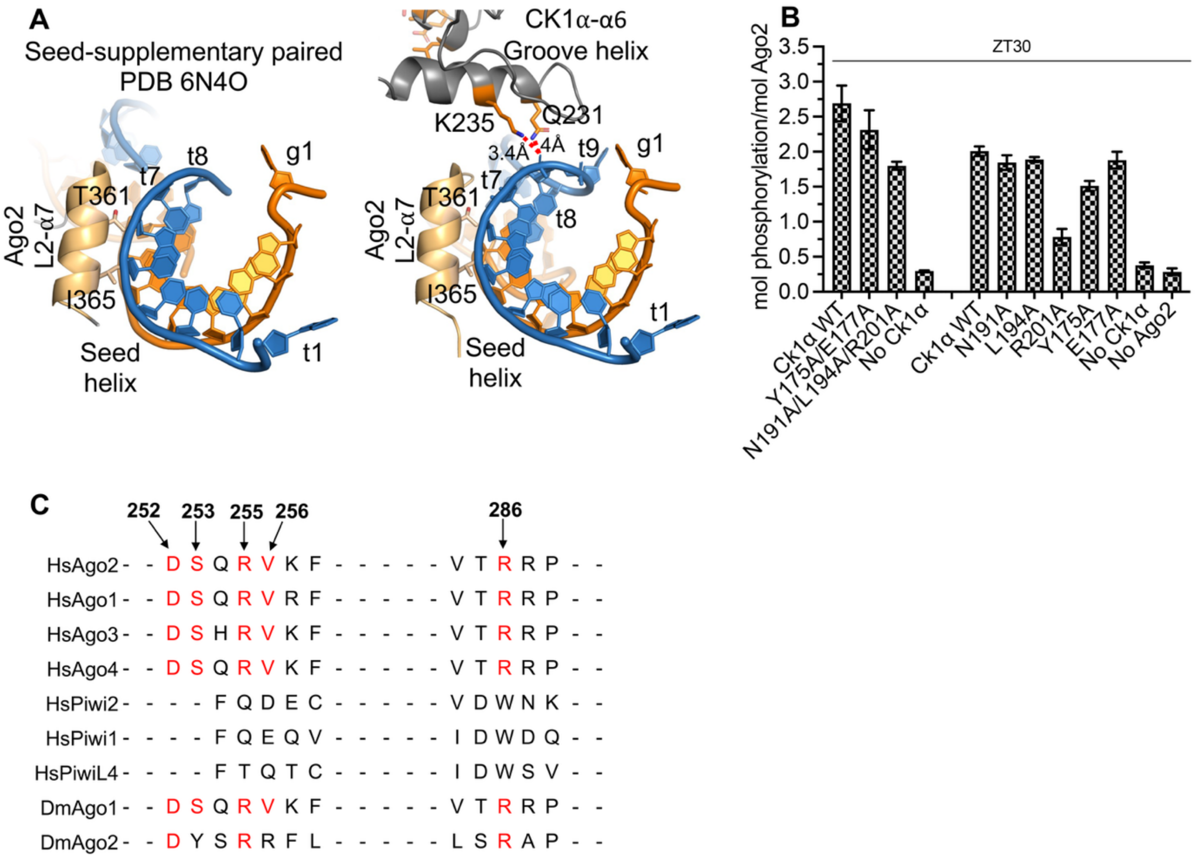

Supplementary Figure 2-

CK1α interactions with RISC^{ZT30}. (A) Side-by-side view of the seed-helix supporting interactions in the Ago2 crystal structure with seed+supplementary region paired (PDB 6N4O) [1] and the RISC^{ZT30}-CK1α structure. CK1α groove-helix (α6) K235, Q231 establishes direct interaction with the guide-target seed-helix. The g6-g7 backbone kink disappears in RISC^{ZT30}. (B) *In vitro* Ago2 phosphorylation assay with ZT30 (checkered pattern) target RNA using different CK1α mutants at the CK1α-PAZ interface. Point mutations in R201 or in cluster mutants significantly reduced Ago2 phosphorylation. (C) Sequence alignment of PAZ domain residues that interact with CK1α in different Ago and Piwi proteins. These residues are completely conserved (red) in all human (Hs) Agos, but not in Piwi proteins or the slicing-specific fly (Dm)Ago2. Residue numbers correspond to HsAgo2.

Supplementary Figure 3-

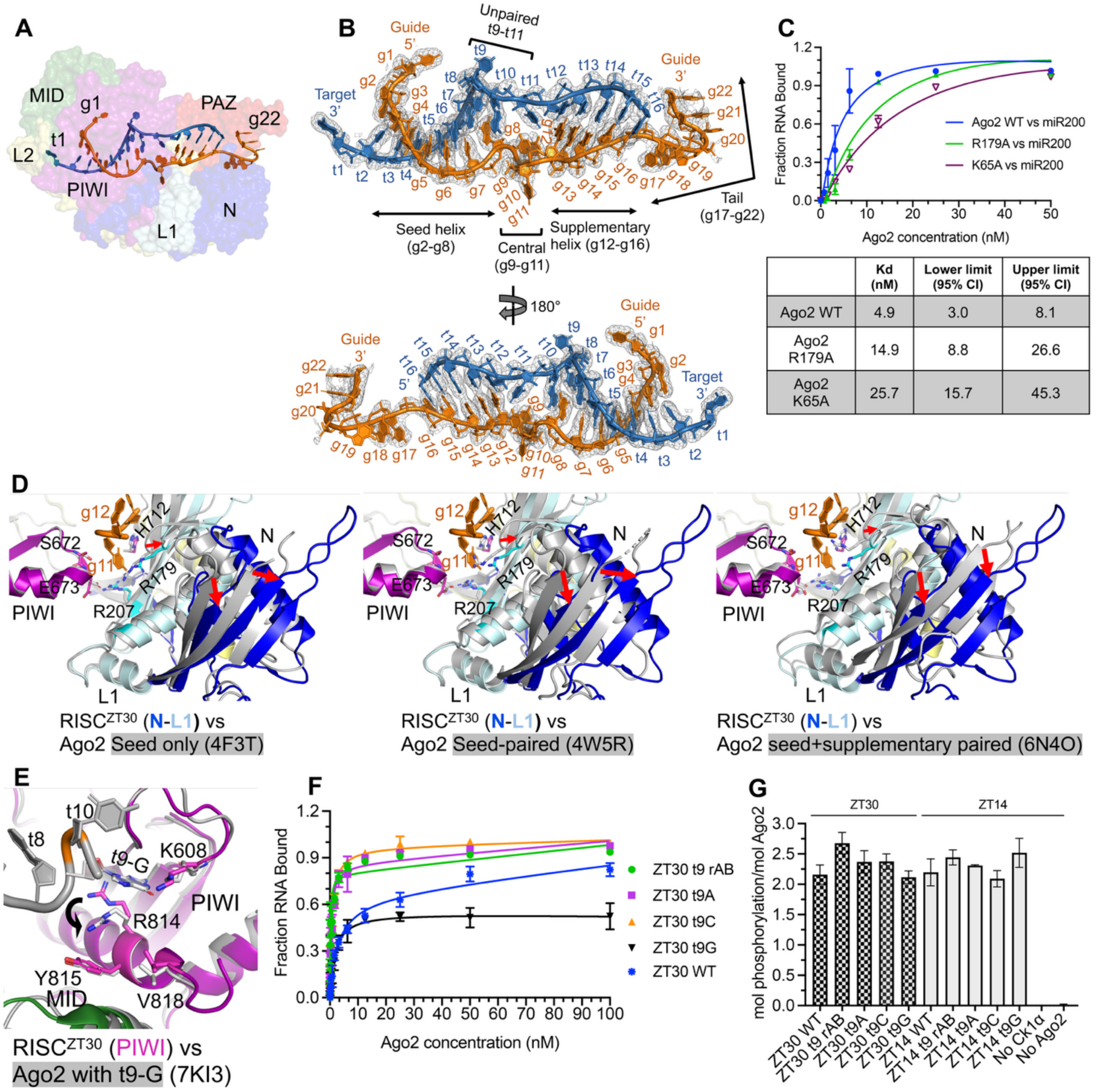

Supplementary Figure 3-

Structural features of guide-target binding in RISC. (A) RISC^{ZT30} structure showing the complete guide-target duplex. (B) The cryoEM map density for the duplex in two different orientations. (C) miR200 guide binding assay for Ago2 with the g11-stacking R179 and g16-stacking K65 mutants. The estimated binding affinities (Kd) with 95% CI limits are also shown (n≥3). (D) A comparative structural analysis showing superimposed g11 (gold)

in N-L1 from RISC^{ZT30} (colored cartoon) with previous Ago2 structures (grey cartoon) containing seed-only (PDB 4F3T) [2], seed-paired (PDB 4W5R) [3], or seed+supplementary paired (PDB 6N4O) [1]. g11 could fit in the same site in other Ago2 structures with appropriate movements in N-L1 domains (highlighted with red arrows). The g11 site is often occupied by R179, and L1 repositioning would open the pocket for g11 binding. (E) A superposition of the RISC^{ZT30} (colored) and a previous Ago2 structure containing a G at the t9 position (grey) (PDB 7KI3) [4]. PIWI R814 would have to reposition in order to fit a t9-G nucleobase (black arrow). Other t9 pocket residues don't move. (F) ZT30 target binding in RISC using t9 RNA variants as indicated. Compared to other ZT30 targets, t9-G RNA shows impaired binding with a significant reduction in the target-bound species at a saturating RISC concentration (100 nM) in filter binding assays (n=3). (G) *In vitro* phosphorylation assay of Ago2 with t9 variants in ZT30 (checkered pattern) and ZT14 (grey) RNAs. Substitution of t9 did not affect Ago2 phosphorylation levels.

Supplementary Figure 4-

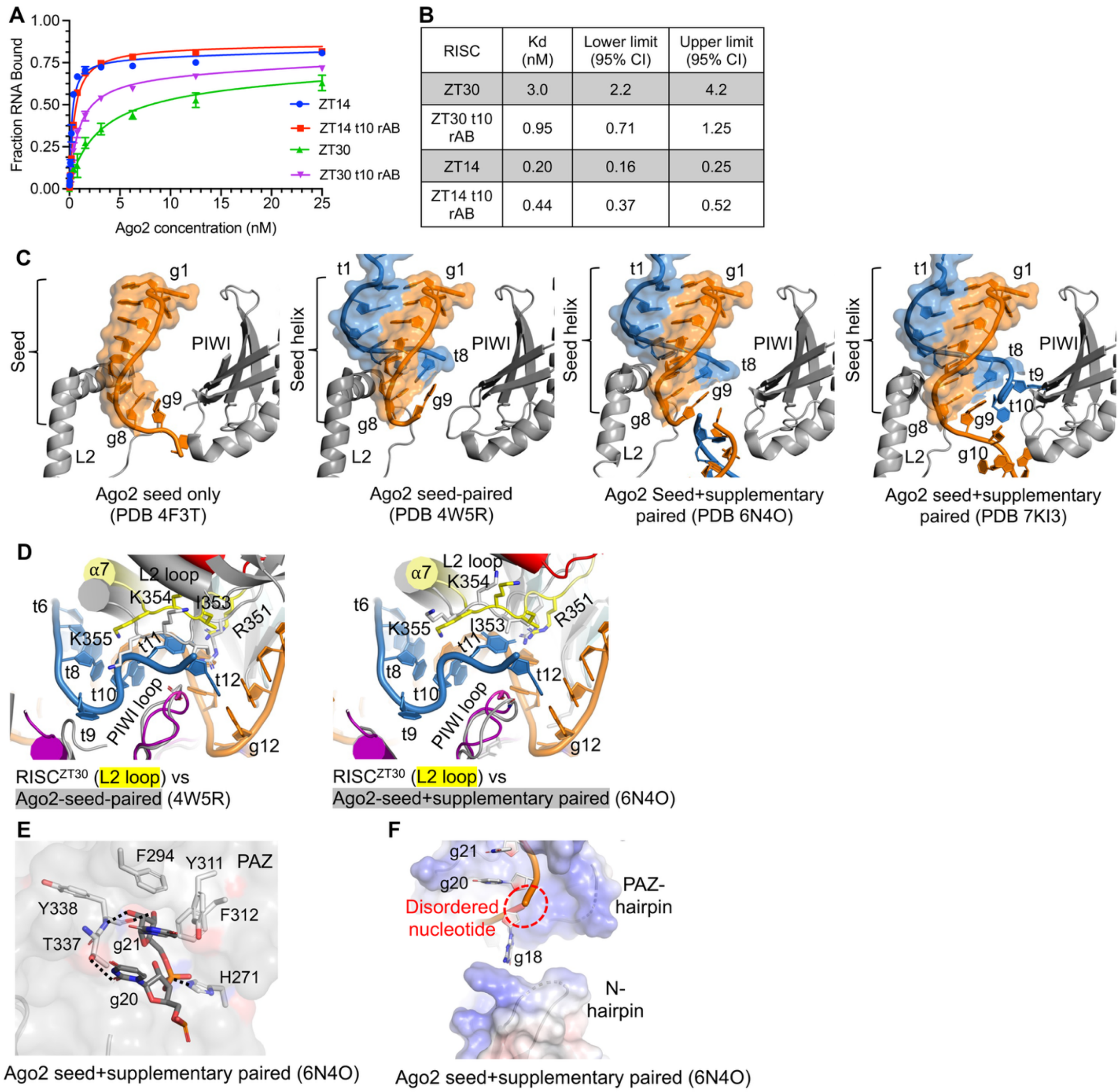

RISC	Kd (nM)	Lower limit (95% CI)	Upper limit (95% CI)
ZT30	3.0	2.2	4.2
ZT30 t10 rAB	0.95	0.71	1.25
ZT14	0.20	0.16	0.25
ZT14 t10 rAB	0.44	0.37	0.52

Supplementary Figure 4-

Features in RISC-target complex assembly. (A) RISC-target binding assays of t10 variants in ZT30 and ZT14 target RNA. (B) Calculated binding affinities (Kd) with 95% CI limits (n=>2). RISC binding with a t10 abasic nucleotide (rAB) does not change binding significantly. (C) Analysis highlighting the g9 positioning in different Ago2 structures containing seed only (PDB 4F3T) [2], seed-paired (PDB 4W5R) [3], seed-supplementary paired (PDB 6N4O and 7KI3) [1,4]. The g9 nucleobase stacks under g8 or the g8-t8 base-pair supporting the seed or seed-helix region. (D) Superposition of the target RNA (blue)

from the RISC^{ZT30} structure on the seed-paired (PDB 4W5R) and seed-supplementary paired (PDB 6N4O) showing that t11 would clash with the L2 loop (R351-K355 region) in those previous Ago2 structures. (E) The 3' tail interaction with PAZ in Ago2 structure (PDB 6N4O). The g20-g21 are tightly bound in the PAZ 3' pocket. The direct H-bonds are highlighted with black dotted lines. (F) The open tail-gate region in the previous Ago2 structure containing seed-supplementary paired region (PDB 6N4O). g19 in this structure remains disordered.

Supplementary Figure 5-

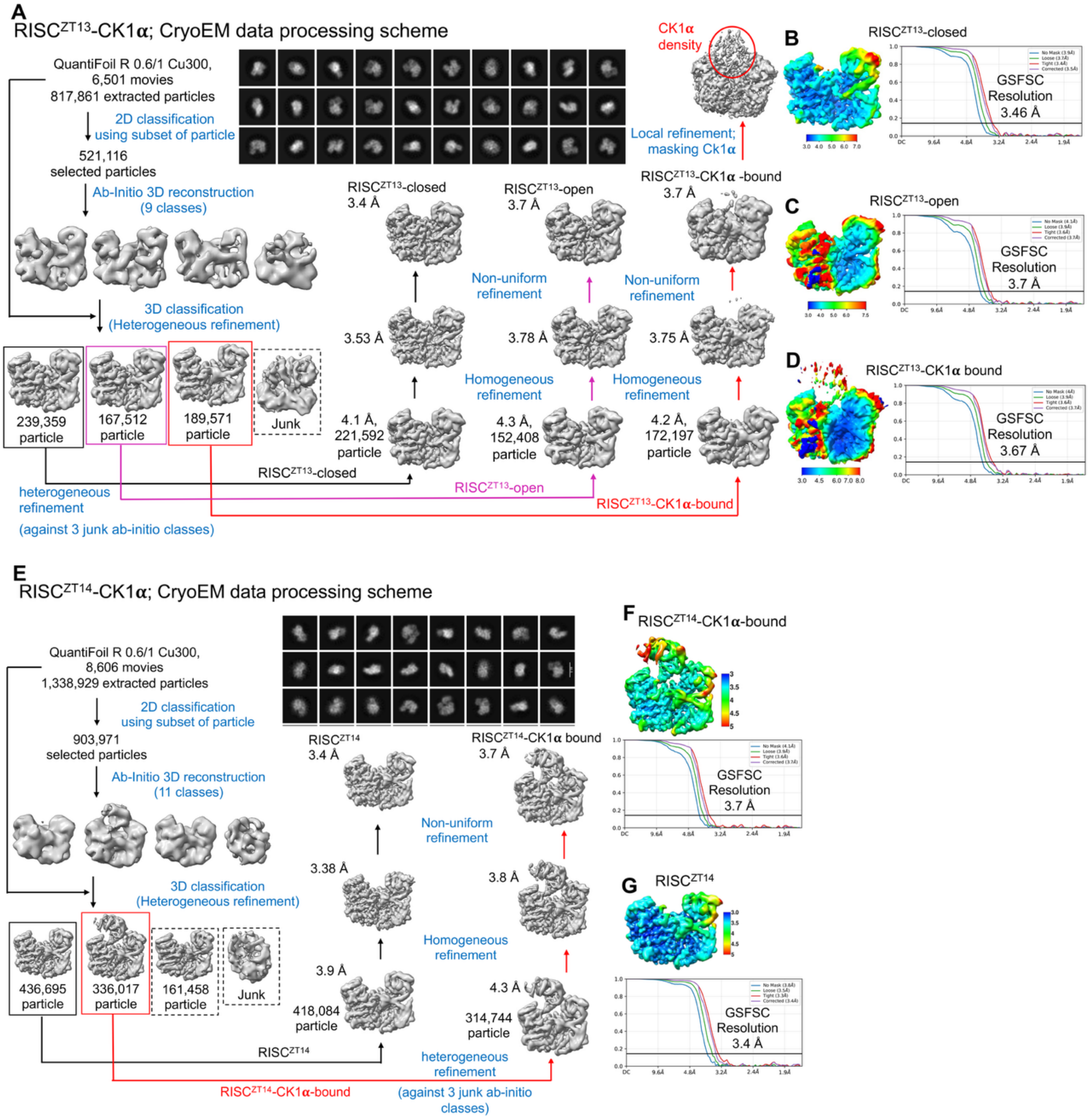

Supplementary Figure 5-

Structural analysis of RISC complexes with shorter targets and CK1α kinase. (A) CryoEM data processing workflow for the RISC^{ZT13}-CK1α complex. Pre-processed cryoEM particles were separated into RISC^{ZT13}-closed, RISC^{ZT13}-open, and RISC^{ZT13}-CK1α states. The observed 2D class averages for some of the classes used in data processing are shown. Local resolution estimates and gold-standard FSC (GSFSC) resolution

estimates for (B) RISC^{ZT13}-closed (C) RISC^{ZT13}-open and RISC^{ZT13}-CK1α maps. (E) CryoEM data processing workflow for the RISC^{ZT14}-Ck1α complex, with 2D average class representatives. The local resolution estimates and gold-standard FSC (GSFSC) resolution estimates for (F) RISC^{ZT14}-Ck1α and (G) RISC^{ZT14} EM maps.

Supplementary Figure 6-

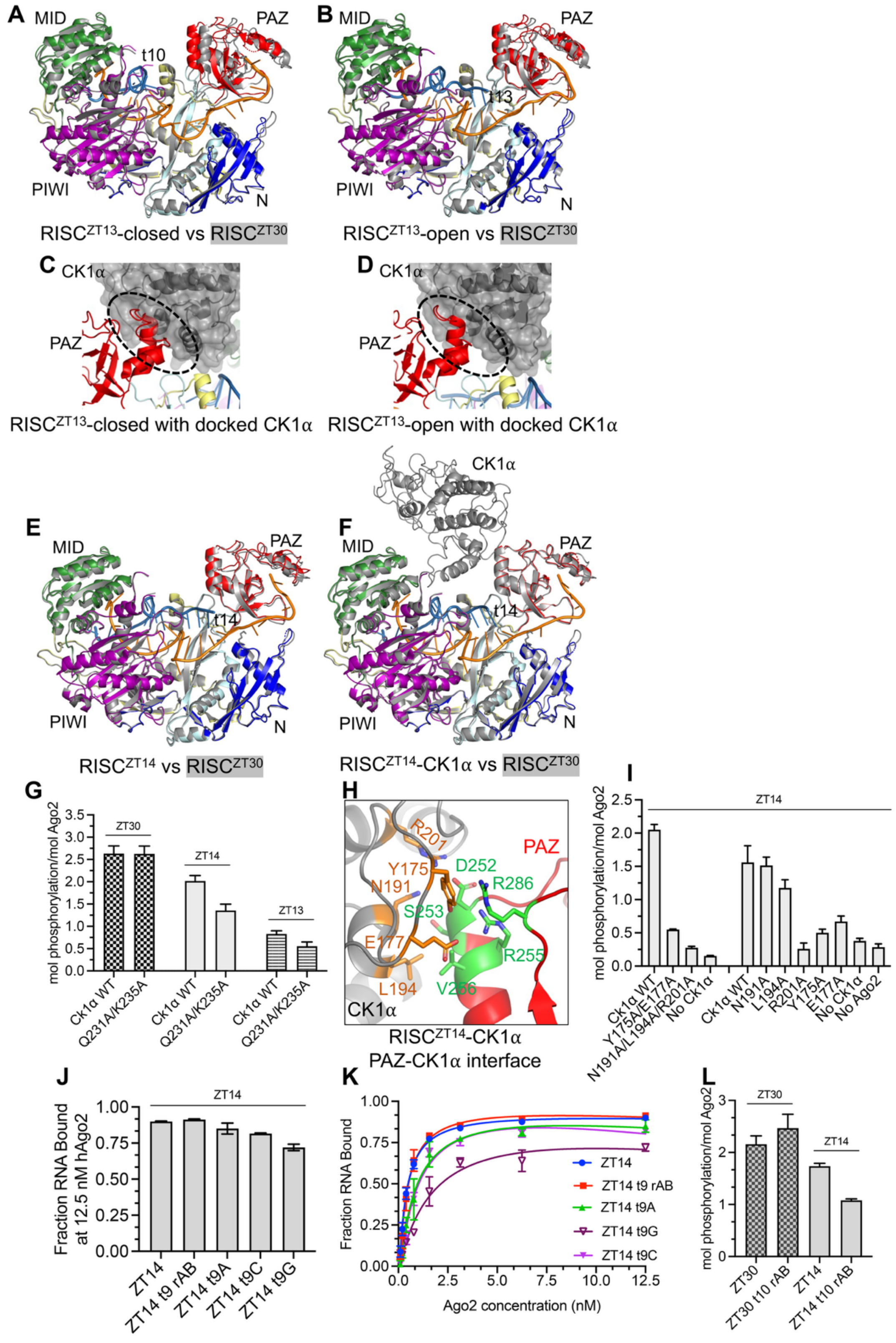

Supplementary Figure 6-

RISC dynamics during CK1α docking. Structural superposition of the (A) RISC^{ZT13}- closed and (B) RISC^{ZT13}-open structures with the RISC^{ZT30} (grey) structure. The PAZ domain leans toward the MID domain in the RISC^{ZT13}-closed structure and widens the RNA binding channel by swinging away from the MID domain. The complete ZT13 target (sky blue) is visible in the open structure, while the RNA in the closed structure is visible only up to the t10 nucleotide. (C) Superimposition of CK1α on the RISC^{ZT13}-closed structure showing clashes at the interface (black dotted circle). (D) Superimposition of CK1α on RISC^{ZT13}-open structure does not show clashes with the PAZ domain (black dotted circle). Superimpositions of (E) RISC^{ZT14} and (F) RISC^{ZT14}-CK1α structures with RISC^{ZT30} (grey). Both RISC^{ZT14} structures superimpose well on RISC^{ZT30} without any clashes between CK1α and PAZ. The complete ZT14 target (sky blue) and miR200 guide (yellow) are visible in both structures. (G) *In vitro* Ago2 phosphorylation assays with CK1α containing groove-helix mutations with different target RNA lengths: ZT30, ZT14, and ZT13. Ago2 phosphorylation is significantly reduced with the ZT14 target but not with other targets. (H) A zoomed-in view of the CK1α-PAZ interface in RISC^{ZT14}-CK1α. All the observed interactions are conserved in the RISC^{ZT30}-CK1α. (I) *In vitro* Ago2 phosphorylation assays using ZT14 (grey bar) target RNA with different CK1α mutations at the CK1α-PAZ interface. Different point mutations show an almost complete loss of Ago2 phosphorylation. (J) The calculated target-bound species at a saturating RISC concentration (12.5 nM) and the (K) traces for the binding assays for different ZT14 t9 RNA variants from filter binding assays (n=3). The t9-G substitution shows only a modest reduction in RISC binding in ZT14 target RNA. (L) *In vitro* Ago2 phosphorylation showing the effect of an abasic nucleotide at t10 (rAB) in the ZT14 and ZT30 targets. t10-rAB ZT14 shows reduced phosphorylation.

Supplementary table-1: data collection and refinement statistics for cryo-EM structure of hAgo2-guide-target-CK1α complexes

**Cryo-EM sample**	**RISC^{ZT13}-CK1α**		**RISC^{ZT14}-CK1α**		**RISC^{ZT30}-CK1α**	
**Data collection and processing**						
Microscope	Titan Krios					
Voltage (kV)	300					
Detector	K3					
Magnification	105,000x					
Pixel size (Å)	0.856					
Data collection software	EPU					
Defocus range (μm)	0.7 – 2.2		0.6 – 2.0		0.6 – 2.2	0.7 – 2.2
Total exposure ($e^-/Å^2$)	75.5		76.65		61.2	67.8
Frames/exposure	30					
Exposure/frame ($e^-/Å^2$)	2.52		2.55		2.04	2.26
Micrographs collected	6,501		8,606		4,328	5,027
Total extracted particles	817,861		1,338,929		820,948	1,126,019
**Structure name**	**RISC^{ZT13}-closed**	**RISC^{ZT13}-open**	**RISC^{ZT14}**	**RISC^{ZT14}-Ck1α**	**RISC^{ZT30}**	**RISC^{ZT30}-Ck1α**
PDB/EMDB	10JF/75215	10JG/75216	10JH/75217	10JI/75218	10JJ/75219	10JK/75220
Final particles	221,592	152,408	418,084	314,744	375,289	487,577
Symmetry	C1					
**Map resolution (unmasked/masked)**						
GSFSC 0.143	3.9/3.5	4.1/3.7	3.8/3.4	4.1/3.7	3.2/2.8	3.1/2.8
**Refinement and model validation**						
**Model resolution (unmasked/masked)**						
FSC 0.143	3.6/3.5	3.8/3.7	3.4/3.3	3.7/3.7	2.8/2.8	2.8/2.7
FSC 0.5	4.1/3.9	4.3/4.2	3.7/3.5	4.1/4.0	3.1/3.0	3.1/3.0
Map CC mask/volume	0.75/0.75	0.69/0.69	0.78/0.78	0.68/0.69	0.85/0.84	0.78/0.78
Protein residue	814	823	823	1111	825	1120
Nucleic acid	32	35	36	36	38	38
Water/Ligand	0/0	0/0	0/0	0/0	0/0	0/1
**B-factors (Å^2)**						
Protein	67.31	67.11	67.09	64.20	67.02	64.08
Nucleotide	68.15	69.65	72.02	72.02	73.28	73.28
**RMS Deviations**						
Bond length (Å)	0.002	0.003	0.003	0.002	0.006	0.006
Bond angle (°)	0.585	0.593	0.536	0.579	0.573	0.642
**Ramachandran statistics**						
Favored (%)	92.29	95.59	96.21	96.73	94.76	95.51
Allowed (%)	7.34	4.41	3.79	3.27	5.24	4.49
Outlier (%)	0.37	0.0	0.0	0.0	0.0	0.0
Rotamer outliers (%)	0.0	0.0	0.0	0.1	0.14	1.01
CaBLAM outliers (%)	4.28	2.59	1.85	1.92	2.33	1.71
Clash score (all atoms)	8.91	10.73	4.66	7.09	4.84	5.67

MolProbity score	1.95	1.86	1.46	1.60	1.60	1.62
FSC is Fourier shell correlation, and RMSD is root-mean-square deviation.						

Supplementary table 2- The sequence of RNA substrates used in the study.

S.no.	Name	Details	Sequence (5'-3')
1	Guide	miR200 (22nt length)	UAAUACUGCCUGGUAAUGAUGA
2	ZT30	30 nt ZT1 target	ACAUUAGCUGAUUUUUACCUAUCAGUAUUA
3	ZT14	14 nt ZT1 target	ACCUAUCAGUAUUA
4	ZT13	13 nt ZT1 target	CCUAUCAGUAUUA
5	ZT30 t9 rAB	30 nt ZT1 target with rAB at t9	ACAUUAGCUGAUUUUUACCUA(rAB)CAGUAUUA
6	ZT30 t9A	30 nt ZT1 target with t9A substitution	ACAUUAGCUGAUUUUUACCUAACAGUAUUA
7	ZT30 t9C	30 nt ZT1 target with t9C substitution	ACAUUAGCUGAUUUUUACCUACCAGUAUUA
8	ZT30 t9G	30 nt ZT1 target with t9G substitution	ACAUUAGCUGAUUUUUACCUAGCAGUAUUA
9	ZT14 t9 rAB	14 nt ZT1 target with rAB at t9	ACC UA(rAB) CAG UAU UA
10	ZT14 t9A	14 nt ZT1 target with t9A substitution	ACCUAACAGUAUUA
11	ZT14 t9C	14 nt ZT1 target with t9C substitution	ACCUACCAGUAUUA
12	ZT14 t9G	14 nt ZT1 target with t9G substitution	ACCUAGCAGUAUUA
13	ZT30 t10 rAB	30 nt ZT1 target with rAB at t10	ACAUUAGCUGAUUUUUACCU(rAB)UCAGUAUUA
14	ZT14 t10 rAB	14 nt ZT1 target with rAB at t10	ACC U(rAB)U CAG UAU UA-OH
15	ZTs	30 nt ZT1 target with full complementarity to miR200 guide	ACAUUAGCUCAUCAUUACCAGGCAGUAUUA
16	g11 rAB	miR200 guide with rAB at g11	UAAUACUGCC(rAB)GGUAAUGAUGA
17	g13/g14 rAB	miR200 guide with rAB at g13/g14 both	UAAUACUGCCUG(rAB)(rAB)AAUGAUGA
18			

The red highlighted nucleotide positions are indicative of either mutations or substitution at that position in RNA sequence.

Supplementary table 3- List of the used reagents, generated plasmids, deposited PDB data and programs used in the study.

Reagent or Resource	Source	Identifier
**Chemicals, peptides and recombinant proteins**		
[γ-^{32}P] ATP	Revvity	Cat# BLU502Z250UC, BLU035C001MC
Strep-Tactin 4flow high-capacity resin	IBA Lifesciences	Cat# 2-1250-025
SYBR Gold Nucleic acid gel stain	ThermoFisher Scientific	Cat# S11494
DSS, No-weight format	ThermoFisher Scientific	Cat# A39267
T4 Polynucleotide Kinase	NEB	Cat# M0201L
Proteinase K	NEB	Cat# P8107S
RNasIn RNase inhibitor	Promega	Cat# N2515
Octyl β-D-glucopyranoside	ThermoFisher Scientific	Cat# BP585-1
HyClone CCM3 media	Cytiva	Cat# SH30065.02
d-Desthiobiotin	Millipore-Sigma	Cat# D1411-1G
HiTrap SP-HP Column	Cytiva	Cat# 17115201
Mono S 10/100 GL Column	Cytiva	Cat# 17516901
Superose 6 increase 3.2/300 column	Cytiva	Cat# 29091598
Superose 6 increase 10/300 column	Cytiva	Cat# 29091596
Superdex 200 increase 10/300 column	Cytiva	Cat# 28990944
Superdex 75 increase 10/300 column	Cytiva	Cat# 29148721
**Deposited data**		
Coordinates for Ago2-miR200b-ZT13 (RISC^{ZT13})-open structure	This work	PDB:10JG

Cryo-EM maps for Ago2-miR200b-ZT13 (RISC^{ZT13})-open structure	This work	EMDB:75216
Coordinates for Ago2-miR200b-ZT13 (RISC^{ZT13})-closed structure	This work	PDB:10JF
Cryo-EM maps for Ago2-miR200b-ZT13 (RISC^{ZT13})-closed structure	This work	EMDB:75215
Coordinates for Ago2-miR200b-ZT14 (RISC^{ZT14}) structure	This work	PDB:10JH
Cryo-EM maps for Ago2-miR200b-ZT14 (RISC^{ZT14}) structure	This work	EMDB:75217
Coordinates for Ago2-miR200b-ZT14 (RISC^{ZT14}) -CK1α structure	This work	PDB:10JI
Cryo-EM maps for Ago2-miR200b-ZT14 (RISC^{ZT14}) -CK1α structure	This work	EMDB:75218
Coordinates for Ago2-miR200b-ZT30 (RISC^{ZT30}) structure	This work	PDB:10JJ
Cryo-EM maps for Ago2-miR200b-ZT30 (RISC^{ZT30}) structure	This work	EMDB:75219
Coordinates for Ago2-miR200b-ZT30 (RISC^{ZT30}) -CK1α structure	This work	PDB:10JK
Cryo-EM maps for Ago2-miR200b-ZT30 (RISC^{ZT30}) -CK1α structure	This work	EMDB:75220

**Experimental models: Cell lines**		
Sf9 insect cells	ThermoFisher Scientific	Cat# 11496015
High Five insect cells	ThermoFisher Scientific	Cat# B85502
E.coli DH5α cells (high efficiency)	ThermoFisher Scientific	Cat# 18258-012
E.coli DH10Bac cells	ThermoFisher Scientific	Cat# 12033-015
E.coli Rosetta 2 (DE3) cells	Novagen	Cat# 71400
**Oligonucleotides**		
RNA sequences. See Table S2.	N/A	N/A
**Recombinant DNA**		
pFL-SST-HsAgo2	Elkayam et al., 2012	N/A
pFL-SST-HsAgo2(R255A/R286A)	This work	N/A
pFL-SST-HsAgo2(D252A/S253A/V256S)	This work	N/A
pFL-SST-HsAgo2(D358A)	This work	N/A
pFL-SST-HsAgo2(R255A)	This work	N/A
pFL-SST-HsAgo2(R286A)	This work	N/A
pFL-SST-HsAgo2(D252A)	This work	N/A
pFL-SST-HsAgo2(S253A)	This work	N/A
pFL-SST-HsAgo2(V256S)	This work	N/A
pFL-SST-HsAgo2(K65A)	This work	N/A
pFL-SST-HsAgo2(R179A)	This work	N/A
pFL-SST-HsAgo2(R179A/K65A)	This work	N/A
pFL-SST-CK1α	Bri et al., 2022	N/A
pFL-SST-CK1α (Y175A/E177A)	This work	N/A
pFL-SST-CK1α (N191A/L194A/R201A)	This work	N/A
pFL-SST-CK1α (Y175A)	This work	N/A
pFL-SST-CK1α (E177A)	This work	N/A

pFL-SST-CK1α (N191A)	This work	N/A
pFL-SST-CK1α (L194A)	This work	N/A
pFL-SST-CK1α (R201A)	This work	N/A
pFL-SST-CK1α (R185A)	This work	N/A
pFL-SST-CK1α (R185A/G223A/K232A)	This work	N/A
pFL-SST-CK1α (R134A)	This work	N/A
λPP-pET28a	Bri et al, 2022	N/A
**Software and algorithms**		
EPU software	ThermoFisher Scientific	
CryoSPARC v2	Punjani et al., 2017	https://cryosparc.com
WARP	Tegunov and Cramer, 2019	http://www.warpem.com/warp/
Coot	Emsley and Cowtan, 2004	https://www2.mrc-lmb.cam.ac.uk/personal/pemsley/coot/
PHENIX 1.20	Adams et al., 2010	http://www.phenix-online.org/
ChimeraX	Pettersen et al., 2004	https://www.cgl.ucsf.edu/chimerax/
PyMol 2.5.5	Schrödinger LLC	https://pymol.org/2/
MolProbity	Chen et al., 2010.	http://molprobity.biochem.duke.edu/
PRISM 9.5.1 software	GraphPad	https://www.graphpad.com/scientific-software/prism/

References

1 Sheu-Gruttadauria, J., Xiao, Y., Gebert, L. F. & MacRae, I. J. Beyond the seed: structural basis for supplementary microRNA targeting by human Argonaute2. *EMBO J* **38**, e101153, doi:10.15252/embj.2018101153 (2019).

2 Elkayam, E. *et al.* The structure of human argonaute-2 in complex with miR-20a. *Cell* **150**, 100-110, doi:10.1016/j.cell.2012.05.017 (2012).

3 Schirle, N. T., Sheu-Gruttadauria, J. & MacRae, I. J. Structural basis for microRNA targeting. *Science* **346**, 608-613, doi:10.1126/science.1258040 (2014).

4 Gebert, L. F. R., Law, M. & MacRae, I. J. A structured RNA motif locks Argonaute2:miR-122 onto the 5' end of the HCV genome. *Nat Commun* **12**, 6836, doi:10.1038/s41467-021-27177-9 (2021).
